# Development of a Rasch-Built Amyotrophic Lateral Sclerosis Impairment Multidomain Scale to Measure Disease Progression in ALS

**DOI:** 10.1212/WNL.0000000000207483

**Published:** 2023-08-08

**Authors:** Adriaan D. de Jongh, Ruben P.A. van Eijk, Leonhard A. Bakker, Tommy M. Bunte, Anita Beelen, Conny van der Meijden, Michael A. van Es, Johanna M.A. Visser-Meily, Esther T. Kruitwagen, Jan H. Veldink, Leonard H. van den Berg

**Affiliations:** From the Department of Neurology (A.D.d.J., R.P.A.v.E., L.A.B., T.M.B., M.A.v.E., J.H.V., L.H.v.d.B.), UMC Utrecht Brain Center, University Medical Center Utrecht; Department of Neurology (A.D.d.J.), Tergooi Hospital, Blaricum; Biostatistics & Research Support (R.P.A.v.E.), Julius Center for Health Sciences and Primary Care, Department of Rehabilitation, Physical Therapy Science and Sports (L.A.B., A.B., J.M.A.V.-M., E.T.K.), UMC Utrecht Brain Center, and Center of Excellence for Rehabilitation Medicine (A.B., J.M.A.V.-M., E.T.K.), UMC Utrecht Brain Center, University Medical Center Utrecht, and De Hoogstraat Rehabilitation; and ALS Patients Connected (C.v.d.M.), Bilthoven, the Netherlands.

## Abstract

**Background and Objectives:**

Current scales used in amyotrophic lateral sclerosis (ALS) attempt to summarize different functional domains or “dimensions” into 1 overall score, which may not accurately characterize the individual patient's disease severity or prognosis. The use of composite score risks declaring treatments ineffective if not all dimensions of ALS disease progression are affected equally. We aimed to develop the ALS Impairment Multidomain Scale (AIMS) to comprehensively characterize disease progression and increase the likelihood of identifying effective treatments.

**Methods:**

The Revised ALS Functional Rating Scale (ALSFRS-R) and a preliminary questionnaire, based on literature review and patient input, were completed online by patients from the Netherlands ALS registry at bimonthly intervals over a period of 12 months. A 2-week test-retest, factor analysis, Rasch analysis, and a signal-to-noise optimization strategy were performed to create a multidomain scale. Reliability, longitudinal decline, and associations with survival were evaluated. The sample size required to detect a 35% reduction in progression rate over 6 or 12 months was assessed for a clinical trial that defines the ALSFRS-R or AIMS subscales as a primary endpoint family.

**Results:**

The preliminary questionnaire, consisting of 110 questions, was completed by 367 patients. Three unidimensional subscales were identified, and a multidomain scale was constructed with 7 bulbar, 11 motor, and 5 respiratory questions. Subscales fulfilled Rasch model requirements, with excellent test-retest reliability of 0.91–0.94 and a strong relationship with survival (*p* < 0.001). Compared with the ALSFRS-R, signal-to-noise ratios were higher as patients declined more uniformly per subscale. Consequently, the estimated sample size reductions achieved with the AIMS compared with those achieved with the ALSFRS-R were 16.3% and 25.9% for 6-month and 12-month clinical trials, respectively.

**Discussion:**

We developed the AIMS, consisting of unidimensional bulbar, motor, and respiratory subscales, which may characterize disease severity better than a total score. AIMS subscales have high test-retest reliability, are optimized to measure disease progression, and are strongly related to survival time. The AIMS can be easily administered and may increase the likelihood of identifying effective treatments in ALS clinical trials.

## Introduction

Amyotrophic lateral sclerosis (ALS) is a heterogeneous, multifaceted neurodegenerative disease with multiple underlying pathophysiologic mechanisms and differential clinical phenotypes.^1,2^ The Revised ALS Functional Rating Scale (ALSFRS-R) is most commonly used to evaluate disease severity, monitor disease progression, and serve as primary endpoint in clinical trials^3^ because it is easy to administer and strongly predictive of survival.^4,5^

The ALSFRS-R is, however, multidimensional, meaning that multiple independent facets of ALS disease progression (“dimensions,” i.e., bulbar, motor, and respiratory functioning) are summarized into 1 total score. The fundamental problem is that patients with equal total scores may not be comparable in their disease severity or prognosis, which complicates the assessment of disease progression and treatment effects.^6-9^ Alternatives have been developed, such as the ALS severity scale and Rasch-Built Overall ALS Disability Scale (ROADS),^10,11^ which similarly summarize different ALS symptoms into 1 total score. A total score may not, however, accurately characterize the disease severity of all different ALS phenotypes.^2,6,9^ Bulbar-onset and spinal-onset patients, for example, have different disease courses,^8^ respiratory insufficiency may occur at any time point, and many patients will never develop bulbar symptoms or weakness in all limbs.^2,12^ In addition, treatment effects measured by a total score can become diluted when treatments do not affect all ALS domains equally.^7^ Analyzing ALSFRS-R subscales separately may characterize disease progression more comprehensively but does not solve inherent measurement problems because many ALSFRS-R item options are never the most probable answer during the course of the disease.^6,10^ Moreover, the ALSFRS-R domains are ordinal instead of linearly weighted, meaning that a 1-point decline can represent either a small or large loss of functional ability depending on the question. Rasch-built scales ensure that weighting is linear and worse answer options progressively become more probable during the course of the disease. In fact, Rasch analysis, combined with longitudinal evaluation of candidate questions, may further improve the development of a more sensitive outcome measure for ALS clinical trials.

To maximize the likelihood of identifying effective treatments and improve the utility of questionnaires to monitor disease progression, alternative scales are needed that account for multidimensionality, satisfy Rasch measurement standards, and maximize changes over time. In this study, therefore, we aimed to develop the ALS Impairment Multidomain Scale (AIMS) to characterize disease progression comprehensively.

## Methods

### Questionnaire Development

A preliminary ALS disability questionnaire was created using literature review, international guidelines for ALS, clinical judgment of a panel of experts, and patient input. The literature review included existing scales and questionnaires that measure ALS function or disability^3,11,13-16^ and guidelines and reviews^17-20^ describing ALS symptoms. The expert panel consisted of 3 neurologists (M.A.v.E., J.H.V., and L.H.v.d.B.) and 2 senior researchers (R.P.A.v.E. and A.B.), all with extensive expertise in ALS and neuromuscular diseases. The aim was to compile a complete set of questions that covers the full range of disease progression and disability levels in ALS. The preliminary questionnaire consisted of 110 questions, each with 5 answer options on a Likert-type scale, similar to the Center for Neurological Study Bulbar Function Scale.^13^ Subsequently, think-aloud interviews were conducted with 7 patients to assess the acceptability, clarity, intelligibility, and completeness of the questionnaire. After completing these interviews, questions were adjusted linguistically if patients did not fully understand them. The final questionnaire was translated into English by a professional interpreter and back translated into Dutch for validation. This Dutch translation was compared with the original Dutch version of the AIMS and checked for inconsistencies. The final English and Dutch versions of the AIMS can be found in eAppendices 1 and 2 (links.lww.com/WNL/C899 and links.lww.com/WNL/C900), respectively.

### Participants

**Figure 1:**
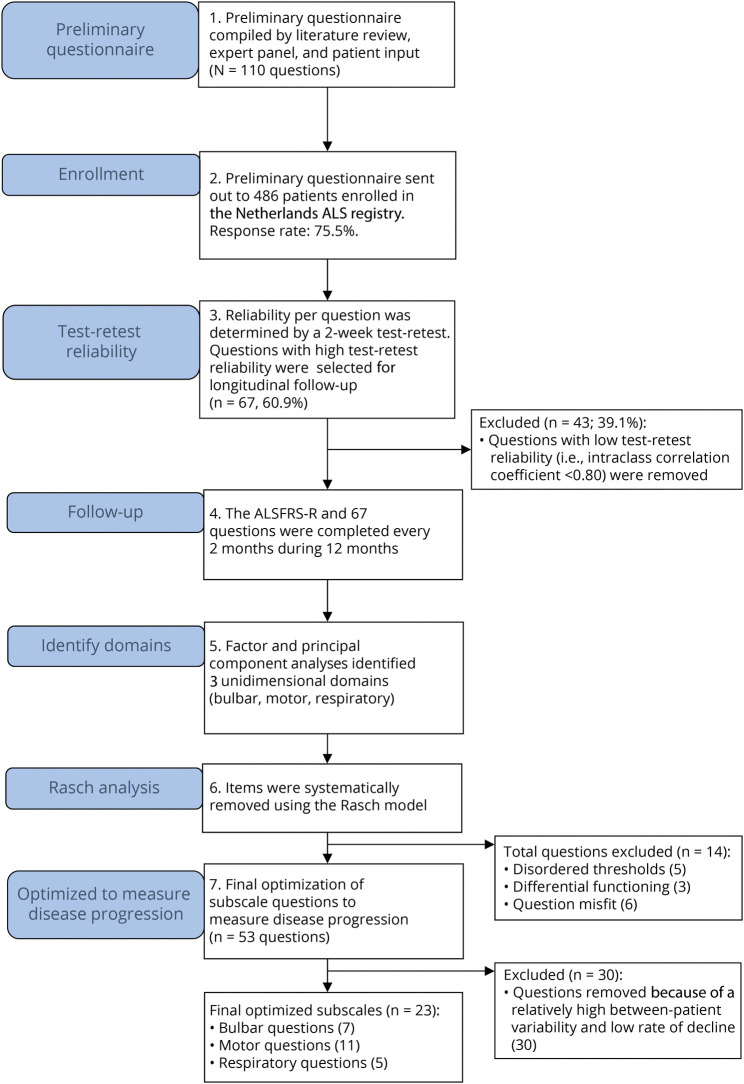
Flowchart of Study Design and Questionnaire Development Steps The flowchart shows the study and questionnaire development steps used in this study. A preliminary questionnaire with 110 questions was sent to patients with ALS enrolled in the Netherlands ALS registry. Test-retest reliability, longitudinal follow-up, Rasch analysis, and optimization to measure disease progression using longitudinal data were performed to create the final unidimensional bulbar, motor, and respiratory subscales of the AIMS. AIMS = ALS Impairment Multidomain Scale; ALS = amyotrophic lateral sclerosis; ALSFRS-R = Revised ALS Functional Rating Scale.

In total, 486 patients with ALS, enrolled in the Netherlands ALS registry, who had previously consented to be approached for research purposes, were sent a link to the preliminary AIMS questionnaire and validated patient-reported version of the ALSFRS-R,^3,4^ through email, on October 11, 2019 (Figure 1). The population-based Netherlands ALS registry has been registering patients with ALS prospectively since 2006; it has been described in detail elsewhere.^21,22^ In brief, patients diagnosed with ALS, according to the revised El Escorial or Gold Coast criteria,^23,24^ were identified through annual screening of hospital registries, through specialized ALS rehabilitation clinic registries, and by contacting neurologists individually. Survival time (defined as the time between enrollment and date of death or date last known to be alive) was obtained for all patients by checking the municipal register at quarterly intervals. As of October 11, 2021, which was the cutoff date for the survival analysis, 167 patients (45.5%) had died and 2 patients (0.54%) with less than 6 months of follow-up time were censored administratively.

### Study Procedures

After completing the preliminary questionnaire, participants received a second link to the preliminary questionnaire to be completed within 14 days to evaluate test-retest reliability per question. Questions with high test-retest reliability (see further) were selected for the longitudinal phase, which required the patients to complete the questions and the validated patient-reported version of the ALSFRS-R^3,4^ every 2 months during 12 months of follow-up. All study data were input and stored in an online database using CASTOR Electronic Data Capture software.^25^

### Reliability and Rasch Analyses

Test-retest reliability was assessed by calculating the intraclass coefficient (ICC) for questions that were completed twice within 14 days; questions with an ICC less than 0.80 were removed. Exploratory factor analysis with varimax rotations was used to identify ALS domains, and model fit was assessed by the root mean square error of approximation (RMSEA); an RMSEA of <0.08 was considered acceptable.^6^ Questions were grouped in subscales according to the pattern of factor loadings. Principal component and factor analyses were performed to evaluate unidimensionality per subscale, which was defined as variance explained by the measured construct of >50%.^26^ For the Rasch analyses, 1 observation per patient was randomly sampled from their longitudinal measurements to avoid dependency in the data. Rasch analyses were performed using the partial credit model,^27^ allowing each question to have its own category probability curves.^28^ Ideally, category probability curves should demonstrate that as disease progresses and disability increases, worse question responses sequentially become more likely. If not, question thresholds are disordered. Category probability curves were examined, and any questions with disordered thresholds were removed. Differential item functioning occurs when different groups of patients with the same overall disability level answer questions significantly differently.^28^ We compared questionnaire responses according to sex, age, and site of symptom onset (i.e., bulbar or spinal onset) using a likelihood ratio test adjusted for multiple testing, and questions that showed significant differential item functioning were removed. Question misfit was evaluated by mean square fit statistics and by comparing the observed proportions with 95% CIs per question response with the predicted probabilities of the Rasch model; misfitting questions, demonstrating significant dependency or unmodeled noise, were removed. Correlations between questions were assessed to avoid interdependent questions.

### Optimization to Measure Disease Progression

Per subscale, longitudinal rates of decline were estimated using linear mixed-effects models with a fixed effect for time and a random intercept and slope for time per patient. The average monthly rate of decline was assessed by the fixed effect of time, whereas between-patient variability was defined as the SD of the random effects for time (i.e., individual progression rates). The signal-to-noise ratio was defined as the ratio between rate of decline and between-patient variability. To allow a direct comparison of between-patient variability in the rate of decline with the ALSFRS-R, scores were standardized by subtracting the mean and dividing by the SD. Of importance, this linear transformation does not affect the ratio between the rate of decline and between-patient variability or the required sample size. Signal-to-noise ratios of the individual bulbar, motor, and respiratory subscales were optimized by minimizing the sample size required to detect a given treatment effect in a clinical trial for all possible combinations of questions^29,30^ and by selecting the combination of questions that leads to the lowest required sample size. The required sample size is obtained through a combination of the rate of decline (i.e., the “signal”) and the within-patient and between-patient variance components (i.e., the “noise”). Sample size calculations were based on 80% power to detect a 35% reduction in rate of decline during 6 or 12 months of follow-up, using monthly follow-up and a 2-sided α of 5%. Sample size calculations were performed in a subset of patients more comparable with common clinical trial populations (defined as “trial-eligible patients”), that is, after exclusion of patients with disease duration >36 months, those older than 80 years, or those with the use of noninvasive ventilation at enrollment.

The final bulbar, motor, and respiratory subscales and question difficulties were reviewed by the expert panel for content validity and clinical utility to measure disability and disease progression. Empirical power of the ALSFRS-R and final AIMS subscales to detect a uniform 35% reduction in rate of decline was estimated, using an analytical strategy that evaluates treatment effects per subscale, before stating whether a treatment is effective, while adjusting *p* values for multiple testing using the Hommel method, as previously described.^7^ Empirical power of the ALSFRS-R and AIMS was estimated by resampling (n = 25,000) longitudinal data of 75 patients per arm with replacement. The average rate of decline of 1 sampled arm was then reduced by 35% to simulate a hypothetical treatment effect, and individual ALSFRS-R and AIMS subscale scores were recalculated. In each resampled dataset, we calculated a *p* value for the between-group difference in the rate of decline measured by the ALSFRS-R and AIMS subscales. ALSFRS-R and AIMS subscales were defined as a primary endpoint family, that is, a statistically significant treatment effect on any one of the subscales was considered a positive trial. Empirical power for the ALSFRS-R and AIMS was defined as the proportion of 25,000 resampled datasets with a statistically significant between-group difference in the rate of decline. To make the results easier to understand, we translated empirical power to required sample size to achieve 80% power.^31^

### Construct Validity

Construct validity was assessed by evaluating the associations of the AIMS subscales with the ALSFRS-R and survival time. Linear mixed-effects models containing the bulbar, motor, and respiratory subscales as dependent variable and the corresponding ALSFRS-R subscales as fixed effects were used to evaluate associations with the ALSFRS-R. Nonlinear relationships were modeled using quadratic fixed effects per ALSFRS-R subscale, and a random slope and intercept were used per patient. Bootstrapping (n = 25,000) was used to estimate 95% CIs. Associations of the subscales score at baseline with survival time were assessed using the Kaplan-Meier estimator and Cox regression.

### Standard Protocol Approvals, Registrations, and Patient Consents

The medical ethics committee and institutional review board of the University Medical Center Utrecht approved this study (reference 19/463), and all participants provided informed consent before participating.

### Data Availability

Anonymized data not published within this article will be shared on request from any qualified investigator.

## Results

### Study Population

An overview of how the questionnaire was developed is given in Figure 1. The preliminary questionnaire, consisting of 110 questions, and the self-reported version of the ALSFRS-R were sent to 486 patients with ALS enrolled in the Netherlands ALS registry; 367 patients (75.5%) provided informed consent and completed at least 1 questionnaire. In total, 2,144 questionnaires were completed during 12 months of follow-up with a mean of 5.8 questionnaires and 9.3 months of follow-up time per patient. Characteristics of the study population are summarized in Table 1. One hundred thirty-nine (37.9%) patients fulfilled the definition of trial eligibility based on a disease duration of less than 36 months, age younger than 80 years, and no use of noninvasive ventilation at enrollment. Trial-eligible patients were slightly younger and had a shorter disease duration and better ALSFRS-R score at inclusion, but a faster decline. The average rate of decline in the ALSFRS-R total score was 0.63 (95% CI 0.56–0.71) points per month for all patients and 1.02 (95% CI 0.88–1.17) points per month for the trial-eligible patients.

**Table 1:**
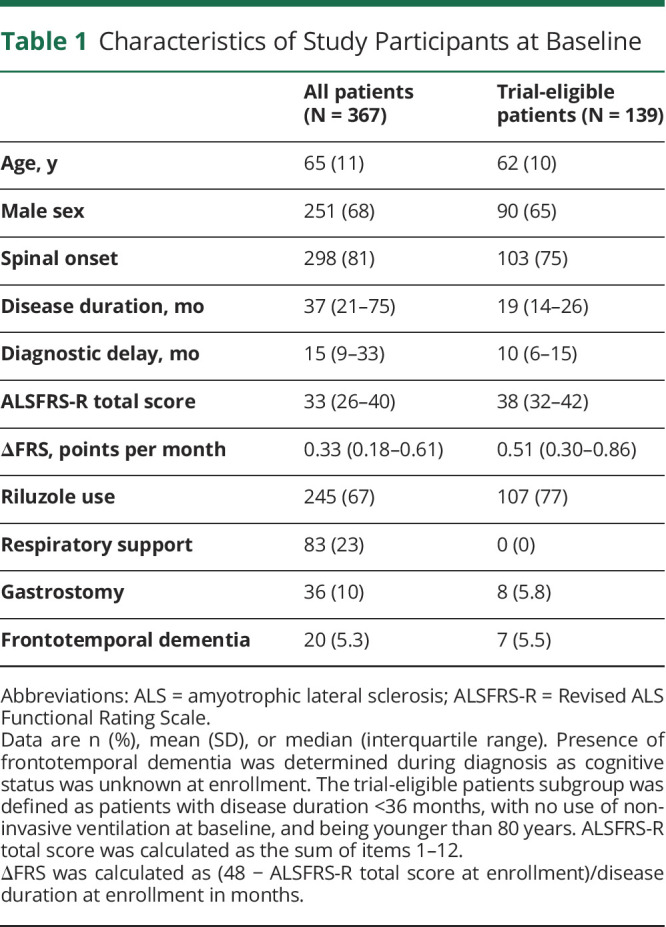
Characteristics of Study Participants at Baseline

The median time to complete the ALSFRS-R and preliminary 120-item questionnaire was 18 minutes (interquartile range 12–30). eFigure 1 (links.lww.com/WNL/C898) shows that all disease stages were represented at all time points because ALSFRS-R total scores ranged from 0 to 48.

### Rasch Analyses

The number of patients who completed a second preliminary questionnaire within 14 days for the test-retest analysis was 146. Of the 110 questions, 67 (61%) had an ICC of 0.80 or higher, thereby surpassing the selection threshold and were subsequently collected during 12 months of follow-up. Principal component and factor analyses found that 3 unidimensional domains were sufficient to explain most of the variance (55.2%) in the data, while this was 49.2% and 59.2% for 2 and 4 domains, respectively. The pattern of factor loadings suggested that questions represented 3 separate domains: a bulbar, a motor, and a respiratory domain.

Category probability curves that describe the probability of each question response per bulbar, motor, and respiratory disability level were examined, and the 3 intermediate answer options were collapsed to resolve disordered thresholds, resulting in a total of 3 response options per question. Five questions (“dietary changes due to swallowing difficulties,” “drooling,” “use of walking aid,” “need help getting out of bed,” and “use of air stacking”) were removed because collapsing response categories did not solve the problem of disordered thresholds. When comparing questionnaire responses between men and women, no significant differential item functioning was observed. Three questions were removed because of differential item functioning; 1 question (“repeating myself to be understood”) demonstrated differential functioning due to age, and 2 questions (“people that understand me tell other people what I said” and “I am aware of my speech disorder”) demonstrated differential functioning on the basis of site of symptom onset. Last, model fit per question was assessed and 6 questions (“in the morning I see saliva on my pillow,” “walking is exhausting,” “standing up,” “changing leg position,” “use of analgesics” and “use of a wheelchair”) were removed because of question misfit.

### Optimization to Measure Disease Progression

**Figure 2:**
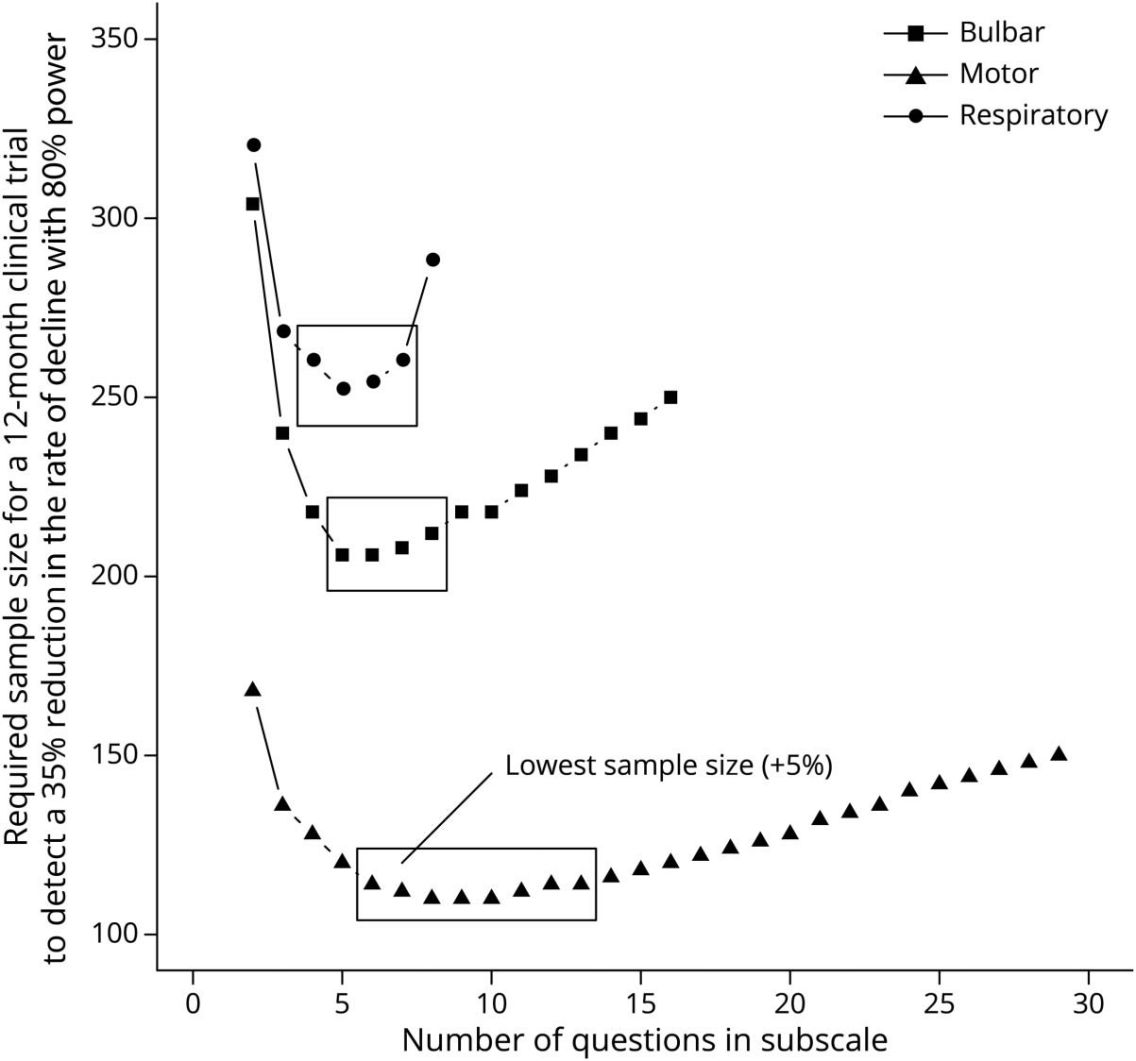
Number of Questions vs Required Sample Size per Subscale The required sample size for a 12-month clinical trial to detect a 35% reduction in progression rate with 80% power was used as an estimate of the sensitivity of candidate subscales to measure disease progression. For each number of questions, the combination of questions that resulted in the lowest required sample size is plotted. Solid rectangles indicate candidate subscales that result in the lowest (+5%) sample size. Initially, as questions are added, information increases, thus reducing the required sample size. However, as more similar questions are subsequently added, between-patient variability in the rate of decline increases, thus inflating the sample size required to detect treatment effects.

**Figure 3:**
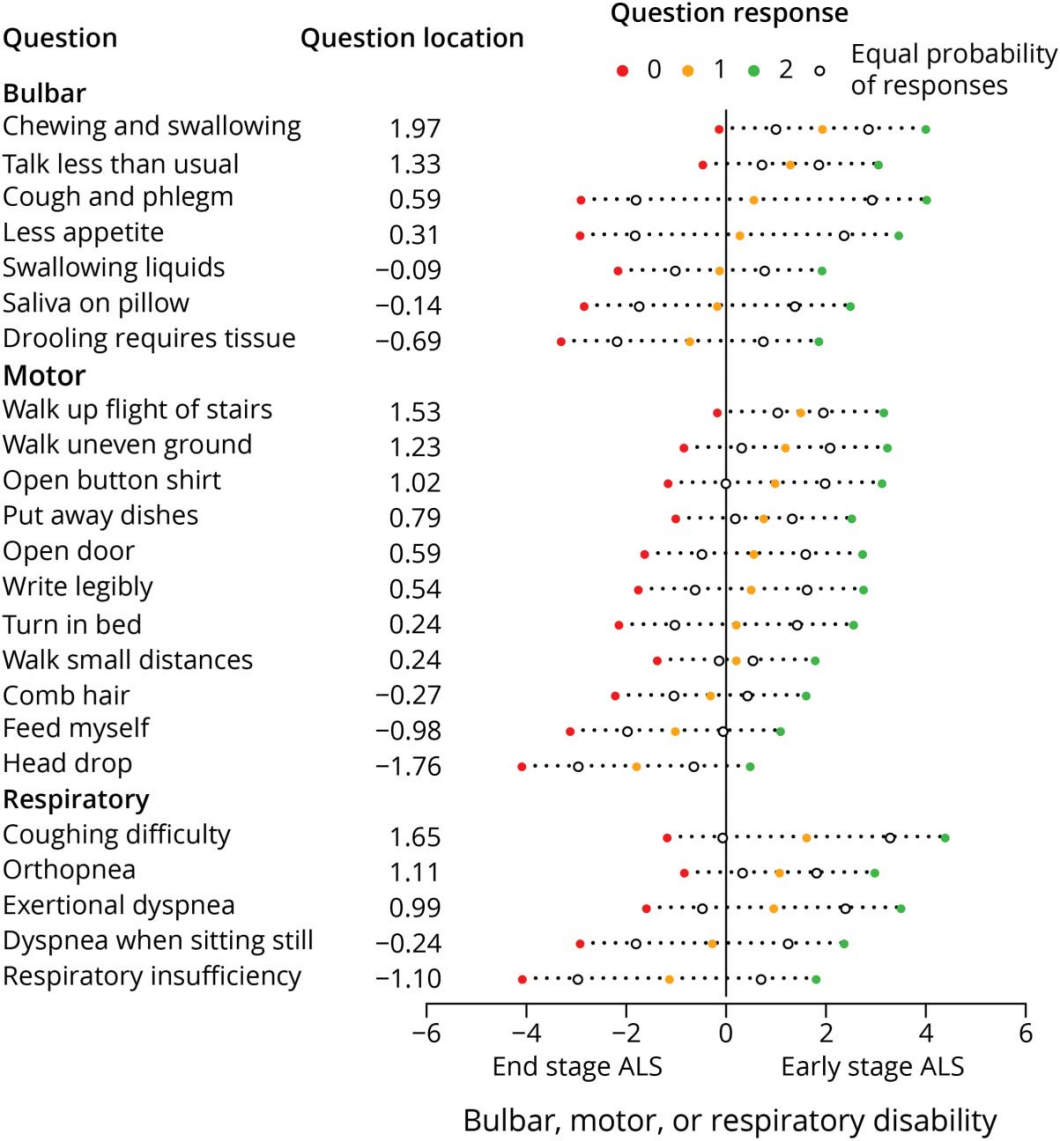
AIMS Question Locations and Thresholds The figure shows the AIMS, question difficulty order, and targeting of each question. The most difficult question (e.g., chewing and swallowing) is on the far right, while the easiest question (e.g., respiratory insufficiency) is on the far left. The x-axis represents the logit measure for a patient's bulbar, motor, or respiratory disability. Worse question options sequentially become more probable as disability increases. AIMS = ALS Impairment Multidomain Scale; ALS = amyotrophic lateral sclerosis.

Bulbar, motor, and respiratory subscales were each optimized to measure ALS disease progression. In total, there were 53 remaining questions that assessed bulbar (n = 14), motor (n = 29), or respiratory (n = 10) functioning. Figure 2 shows the relationship between the total number of questions per subscale and the required sample size. Initially, adding more questions increases information (i.e., increases the average rate of decline, reduces between-patient variability, reduces within-patient variability, or a combination of the 3), thus reducing the sample size required to detect a given treatment effect in a clinical trial. However, at some point, an optimum is reached, where adding more questions does not lead to an increase in information but increases “noise” and hence to an increase in the sample size required to detect treatment effects. The final combination of questions that resulted in the lowest required sample size (or within 5% of the minimum) consisted of 7 bulbar, 11 motor, and 5 respiratory questions. Compared with the ALSFRS-R subscales, the AIMS subscales reduced the 12-month sample size by 23.9%, 27.6%, and 53.6% (Table 2). Next, we estimated the sample size reductions for a clinical trial that defines the ALSFRS-R or AIMS subscales as a primary endpoint family, that is, by evaluating treatment effects univariately per bulbar, motor, and respiratory subscale while adjusting *p* values for multiple testing, before determining whether a treatment is effective overall. In this case, a statistically significant treatment effect on any one of the subscales was considered a positive trial. Compared with the ALSFRS-R subscales, estimated sample size reductions were 16.3% and 25.9%, respectively, for a 6-month and 12-month clinical trial. Results were similar in sensitivity analyses that included patients less comparable with common trial populations (i.e., including patients with disease duration >36 months, being older than 80 years, or the use of noninvasive ventilation at baseline) (eTable 1, links.lww.com/WNL/C898). Question difficulties are presented in Figure 3, showing that worse question options sequentially become more probable as disability per subscale increases. Of importance, the AIMS targeted a broader range of ALS disability levels than the ALSFRS-R, with larger question location disability ranges. Question locations, expressed as logits on a Rasch disability scale, ranged from −0.69 to 1.97, −1.76 to 1.53, and −1.10 to 1.65, for bulbar, motor, and respiratory subscales, respectively, while the corresponding ALSFRS-R subscale question location ranges were 0.18 to 0.94, −0.18 to 1.37, and −0.78 to 0.42.

**Table 2:**
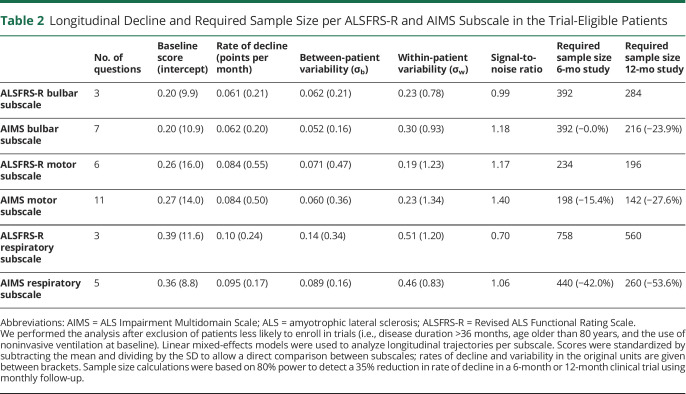
Longitudinal Decline and Required Sample Size per ALSFRS-R and AIMS Subscale in the Trial-Eligible Patients

### Reliability and Construct Validity

**Figure 4:**
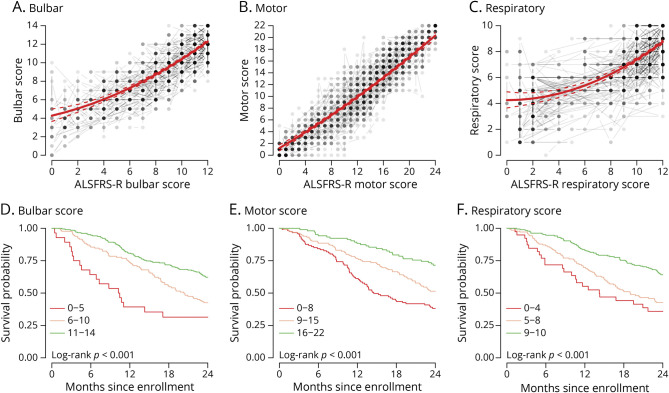
Association of AIMS Subscales With the ALSFRS-R and Survival Time Bulbar, motor, and respiratory AIMS scores were strongly associated with the corresponding ALSFRS-R subscales (A–C) and survival time (D–F). There was a dose-response association between baseline scores and overall survival, lower scores resulting in lower survival probabilities after enrollment. AIMS = ALS Impairment Multidomain Scale; ALS = amyotrophic lateral sclerosis; ALSFRS-R = Revised ALS Functional Rating Scale.

Test-retest reliability for bulbar, motor, and respiratory subscale scores was high with ICCs of 0.94 (95% CI 0.91–0.95), 0.94 (95% CI 0.92–0.96), and 0.91 (95% CI 0.88–0.94), respectively. Finally, to assess construct validity, we evaluated the AIMS subscale score associations with corresponding ALSFRS-R subscales and survival time after enrollment (Figure 4). Correlations with respective ALSFRS-R bulbar, motor, and respiratory subscores were 0.87 (95% CI 0.85–0.90), 0.93 (95% CI 0.92–0.94), and 0.79 (95% CI 0.75–0.82). Compared with the ALSFRS-R, ceiling and floor effects of the AIMS seemed to be smaller. For example, a patient with an ALSFRS-R bulbar score of 0 has, on average, an AIMS bulbar score of 4 (Figure 4A). AIMS subscales were associated with overall survival, lower scores resulting in lower survival probabilities after enrollment, all subscales *p* < 0.001. Or, using Cox regression, hazard ratios for bulbar, motor, and respiratory subscales were 0.90 (95% CI 0.86–0.94), 0.94 (95% CI 0.92–0.97), and 0.84 (95% CI 0.79–0.90), respectively, all *p* < 0.001. Or in other words, a 1-point increase in bulbar, motor, or respiratory score was associated with a 10%, 6%, and 16% reduction in risk of death, respectively.

## Discussion

In this study, we developed the ALS Impairment Multidomain Scale, consisting of unidimensional subscales with 7 bulbar, 11 motor, and 5 respiratory questions, which have high test-retest reliability, fulfill Rasch requirements, and are strongly related to survival time. We optimized subscales by selecting questions that optimize longitudinal rate of decline, while reducing between-patient variability. We have thus developed an easily administered outcome measure for use in ALS clinical trials and in the clinic, which may be more sensitive with a broader measurement window than the ALSFRS-R for monitoring disease progression and detecting treatment effects.

Of importance, by developing a multidomain scale, we aimed to address the multidimensional nature of ALS symptoms. Guidance for regulatory agencies suggests that for some disorders, multiple endpoints may be required for full characterization of the disease.^32^ Multidimensionality is a feature of many neurologic diseases, such as multiple sclerosis,^33^ spinal muscular atrophy,^34^ Parkinson disease,^35^ stroke,^36^ and Alzheimer disease.^37^ Current scales in ALS, such as the ALSFRS-R,^3^ ALS severity scale,^11^ or ROADS,^10^ summarize a range of ALS symptoms in 1 composite score, which may not accurately characterize a multidimensional disease such as ALS. Due to a heterogeneous clinical presentation and different subphenotypes, patients with the same ALSFRS-R total score are not necessarily comparable regarding disease severity, progression rate, or prognosis.^6,8,38^ Moreover, using a composite total score as primary endpoint in clinical trials can disguise important treatment clues. Treatments may not affect all ALS domains equally; as a result, treatment effects measured by composite endpoints may become diluted. For example, in the Nuedexta trial,^39^ treatment improved only bulbar functioning (*p* = 0.003); this treatment effect may have been missed if the ALSFRS-R total score (*p* = 0.25) was defined as the primary endpoint. Obviously, in this study, the beneficial effect of Nuedexta on bulbar functioning was hypothesized beforehand, but the effect is often not known a priori. Similarly, in the edaravone and sodium phenylbutyrate-taurursodiol trials, the treatment effect on the ALSFRS-R total score seemed to be primarily driven by the motor subscale rather than bulbar and respiratory subscales.^40,41^ Likewise, studies that focus on nondrug interventions, such as optimizing multidisciplinary care through physical therapy, exercise programs or speech therapy may also benefit from a more comprehensive assessment of the effects of the intervention.^42^ The multidomain AIMS may, therefore, better characterize ALS disease progression and treatment effects and subsequently facilitate disease monitoring in both the clinic and in trials.

**Figure 5:**
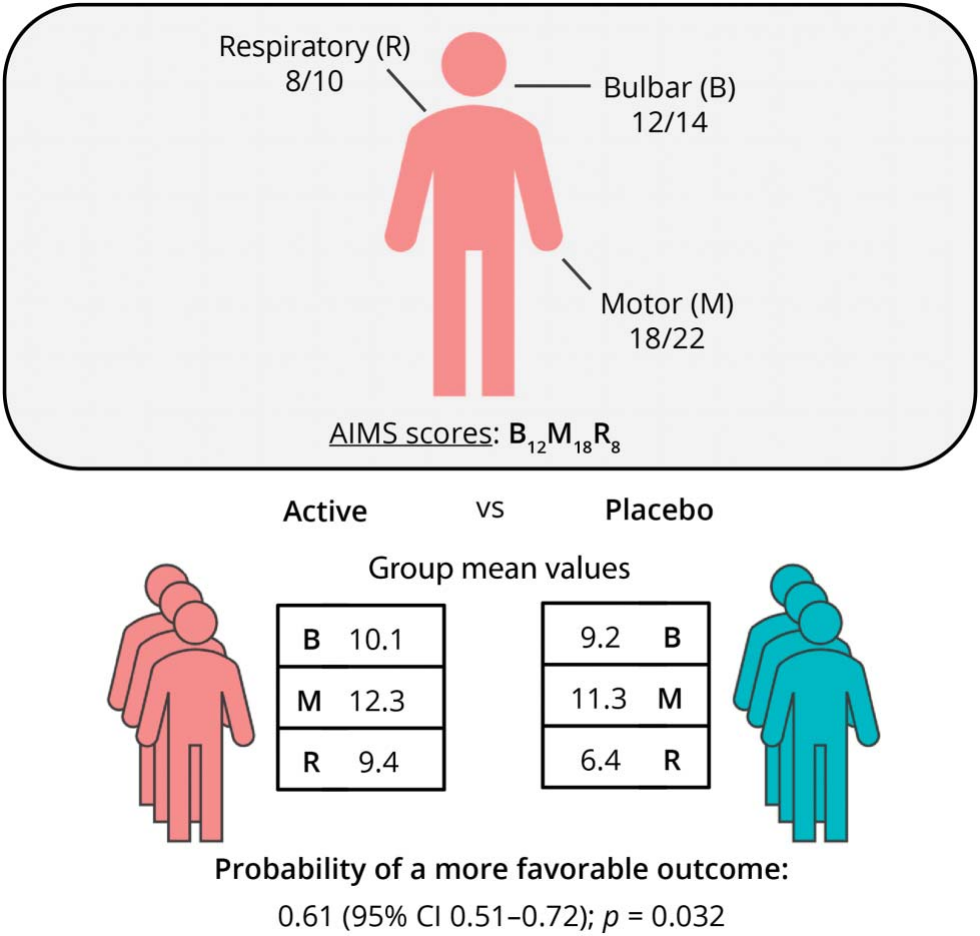
Example Reporting of the AIMS in ALS Clinical Studies In this example study, the AIMS subscales were used as primary endpoint. By weighing each of the domains, for example, according to patient, physician, or prognostic preferences, one can obtain 1 overall effect size with 1 overall *p* value. A similar approach is used by the Combined Assessment of Function and Survival, thereby prioritizing one outcome (survival time) over the other (ALSFRS-R). This approach results in 1 overall effect size; in this case, the probability or odds of having a better outcome when receiving the new therapy compared with that when receiving placebo. Other strategies for analyzing multiple domains are summarized in eTable 3 (links.lww.com/WNL/C898). AIMS = ALS Impairment Multidomain Scale; ALS = amyotrophic lateral sclerosis; ALSFRS-R = Revised ALS Functional Rating Scale.

There are several analytical strategies for analyzing multidomain scales that avoid the pitfalls of composite endpoints, while controlling the false-positive rate (i.e., type I error).^43^ A relatively straightforward method is to evaluate treatment effects per subscale, before stating whether a treatment is effective overall, while adjusting *p* values for multiple testing.^7^ Depending on the investigator's preference, AIMS subscales can a priori be defined as a primary endpoint family,^32^ that is, a treatment effect on any one of the subscales is considered clinically relevant and may be indicative of treatment effectiveness (eTable 2, links.lww.com/WNL/C898, summarizes a worked example). Another option is to rank the importance of each AIMS subscale. This can be performed on a group level (e.g., bulbar is always more important than motor function)^44^ or using individual patient or physician preferences.^45^ The treatment effect can subsequently be summarized as the probability of obtaining a more favorable outcome when treated compared with when receiving placebo. Figure 5 illustrates how this type of analysis could be presented for the AIMS. Nevertheless, as summarized in eTable 3, more complex analytical strategies exist, such as a prespecified testing hierarchy or using multivariate models.^46,47^ Multivariate models simultaneously model multiple longitudinal outcomes, allowing the calculation of 1 *p* value for the overall treatment effect, which may be a more powerful strategy than performing multiple independent statistical tests. Multivariate models are flexible because longitudinal outcomes can be added (e.g., vital capacity, muscle strength, or biomarkers) and, importantly, could be adjusted for mortality.^46,48^

Rasch analysis was used to systematically reduce the number of questions in the preliminary questionnaire to ensure that the AIMS is linearly weighted and that patients can be compared using subscale scores because only 1 concept is being measured (i.e., bulbar, motor, or respiratory disability). To further optimize the multidomain scale, we analyzed longitudinal decline during 12 months of follow-up and selected the combination of questions that results in a high average rate of decline (i.e., “the signal”) with minimal variability (i.e., “the noise”).^29,30^ Consequently, compared with the corresponding ALSFRS-R subscales, the signal-to-noise ratio was improved (Table 2), thus increasing the precision of the AIMS in monitoring disease progression. Reducing variability in trial endpoints is important because it increases precision in estimating treatment effects and reduces the sample size required to detect a given treatment effect.

This study has several limitations that need to be considered. First, cognitive impairment was not evaluated in our study. It is, therefore, not clear whether and to what extent cognitive impairment influenced results for the ALSFRS-R and AIMS. Second, examination of category probability curves indicated collapsing intermediate question responses was necessary to avoid disordered thresholds, making prospective validation of the newly phrased questions necessary. Third, in this study, the AIMS was patient reported, making it easy to incorporate as a remote survey in clinical trials. We have, however, further refined the AIMS by providing guidance per question on when to score 0, 1, or 2. We hypothesize that providing such guidance in combination with adequate training of research personnel may reduce variability in responses within and between patients. However, whether providing guidance and training for scoring indeed reduces variability should be investigated in future studies. Last, we found strong associations of the AIMS subscales with survival time. However, for a questionnaire to be regarded as a true surrogate endpoint for survival time, it is important that a treatment effect on survival time is reflected by the surrogate endpoint and vice versa. Future prospectively designed studies, that, for example, use the joint modelling framework,^46,48^ are, therefore, important in establishing the relationship between treatment effects on the AIMS, ALSFRS-R, and survival time.

In conclusion, we have developed the AIMS, consisting of unidimensional bulbar, motor, and respiratory subscales that may characterize disease severity better than a total score. AIMS subscales have high test-retest reliability, are optimized to measure disease progression, and are strongly related to survival time. The AIMS can be easily administered and may increase the likelihood of identifying effective treatments in ALS clinical trials.

## Glossary

AIMS: ALS Impairment Multidomain Scale
ALS: amyotrophic lateral sclerosis
ALSFRS-R: Revised ALS Functional Rating Scale
ICC: intraclass coefficient
RMSEA: root mean square error of approximation
ROADS: Rasch-Built Overall ALS Disability Scale
